# DEXTER: Disease-Expression Relation Extraction from Text

**DOI:** 10.1093/database/bay045

**Published:** 2018-05-30

**Authors:** Samir Gupta, Hayley Dingerdissen, Karen E Ross, Yu Hu, Cathy H Wu, Raja Mazumder, K Vijay-Shanker

**Affiliations:** 1 Department of Computer and Information Sciences, University of Delaware, 18 Amstel Avenue, Newark, DE 19716, USA; 2 Department of Biochemistry and Molecular Medicine, The George Washington University, Ross Hall, 2300 Eye Street N.W., Washington, DC 20037, USA; 3 Department of Biochemistry and Molecular and Cellular Biology, Georgetown University Medical Center, 3300 Whitehaven St. NW, Suite 1200 Washington, DC 20007, USA; 4 Center for Bioinformatics and Computational Biology, University of Delaware, 15 Innovation Way, Suite 205 Newark, DE 19711, USA

## Abstract

Gene expression levels affect biological processes and play a key role in many diseases. Characterizing expression profiles is useful for clinical research, and diagnostics and prognostics of diseases. There are currently several high-quality databases that capture gene expression information, obtained mostly from large-scale studies, such as microarray and next-generation sequencing technologies, in the context of disease. The scientific literature is another rich source of information on gene expression–disease relationships that not only have been captured from large-scale studies but have also been observed in thousands of small-scale studies. Expression information obtained from literature through manual curation can extend expression databases. While many of the existing databases include information from literature, they are limited by the time-consuming nature of manual curation and have difficulty keeping up with the explosion of publications in the biomedical field. In this work, we describe an automated text-mining tool, Disease-Expression Relation Extraction from Text (DEXTER) to extract information from literature on gene and microRNA expression in the context of disease. One of the motivations in developing DEXTER was to extend the BioXpress database, a cancer-focused gene expression database that includes data derived from large-scale experiments and manual curation of publications. The literature-based portion of BioXpress lags behind significantly compared to expression information obtained from large-scale studies and can benefit from our text-mined results. We have conducted two different evaluations to measure the accuracy of our text-mining tool and achieved average *F*-scores of 88.51 and 81.81% for the two evaluations, respectively. Also, to demonstrate the ability to extract rich expression information in different disease-related scenarios, we used DEXTER to extract information on differential expression information for 2024 genes in lung cancer, 115 glycosyltransferases in 62 cancers and 826 microRNA in 171 cancers. All extractions using DEXTER are integrated in the literature-based portion of BioXpress.

Database URL: http://biotm.cis.udel.edu/DEXTER

## Introduction

Genes contain the information needed to create proteins and dictate cell function; however, it is the pattern of gene expression that determines cell phenotype. Gene expression is highly dynamic and varies widely in different tissues, environmental conditions and disease states. Transcription is controlled by a complex interplay of activators, repressors and chromatin remodeling factors and disruptions in the transcriptional program are well-recognized as drivers of disease (1). Moreover, the critical role played by miRNAs, small RNAs that post-transcriptionally regulate the expression of their target genes, has become increasingly apparent in recent years (2) and abnormalities in miRNA expression have been associated with many diseases (3–8). Identifying genes and miRNAs whose expression levels can guide disease diagnosis, assess prognosis or predict response to therapy is a key aspect of precision medicine (9).

The development of microarray and next-generation sequencing technologies has led to an abundance of transcriptome-wide gene expression data. Much of this data is publicly available through general repositories such as Gene Expression Omnibus (GEO) (10) and Array Express (11), as well as through more specialized resources, such as International Cancer Genome Consortium (ICGC) (12) and the Cancer Genome Atlas (13) (TCGA: http://cancergenome.nih.gov/), which focus on cancer data, and Tissue-specific Gene Expression and Regulation (TiGER) (14), which organizes gene expression data by tissue type. High throughput mass-spectrometry (MS) is providing expression data at the protein level. This data is captured in resources such as dbDEPC (15, 16) a database containing over 4000 differentially expressed proteins in 20 cancers, obtained from 331 MS experiments. While these datasets provide insights into the biological processes and pathways that are affected by changes in the gene/protein expression profile, they are notoriously noisy and so are of limited utility for assessing the behavior of individual genes or proteins.

The scientific literature is a rich source of information on specific gene expression–disease relationships that have been observed in thousands of small-scale studies. In general, these results are only accessible through laborious manual curation; however, automated text-mining tools are beginning to lower the barriers to systematically capturing this data. Several resources focus on manually curated data from publications on disease-related gene and miRNA expression. DisGeNET (17, 18) is a comprehensive platform on human genotype–phenotype relationships, which integrates data from expert curated databases with information gathered through text-mining the scientific literature. miR2Disease (19) is a manually curated database that aims to provide a comprehensive resource for microRNA dysregulation in various human diseases based on published literature. OncomiRDB (20) is a database of experimentally validated cancer-related microRNAs manually curated from literature. miRCancer (21) provides microRNA expression profiles in various human cancers that are extracted from the literature and further confirmed by curators.

Finally, the BioXpress (22, 23) database was developed to address the need for an integrated view of cancer gene and miRNA expression data from a variety of studies, both large and small-scale. BioXpress collects expression data from publicly available sources such as TCGA (13) and ICGC (12), and uses a standardized statistical method to identify the significance of differential expression of genes and microRNAs between tumor and adjacent non-tumor samples from the same patient. In addition, BioXpress reports differential expression of genes manually extracted and curated from publications and Supplementary material, which enables researchers and clinicians to easily compare patients’ expression data with existing knowledge from literature. While there is substantial expression information obtained from large-scale studies in BioXpress (18 626 genes and 710 microRNAs from 33 cancer types and 667 patients), manually curated annotations based on information from the literature (138 genes-PMID annotations) lag behind significantly. Incorporation of automated text-mining tools has the potential to streamline and accelerate the BioXpress curation process.

In this work, we describe an automated text-mining tool, DEXTER to extract information on gene/microRNA expression in the context of diseases. DEXTER extracts the gene or miRNA, the associated disease, the expression level (e.g. high or low), the experimental context (e.g. tissue or cell line) and the conditions being compared. DEXTER’s text-mined results can be used to extend expression databases such as BioXpress, miR2Disease and dbDEMC (24, 25). However, for BioXpress additional constraints needs to be considered as BioXpress reports only differential expression of genes and microRNAs between tumor and normal (non-tumor) samples. Thus, given BioXpress criteria, DEXTER’s extractions will be included in its literature-based portion of the database in cases where tumor and normal tissues are being compared.

One of the motivations in developing DEXTER was to extend the BioXpress database. In fact, DEXTER has been used for three use cases [lung cancer, glycosyltransferase (GT) genes and microRNA related abstracts], with the results having been integrated in literature-based portion of BioXpress (https://hive.biochemistry.gwu.edu/bioxpress/about). We ran DEXTER on 88 431, 27 516 and 28 067 abstracts for lung cancer, GT and microRNAs related abstracts, respectively. Differential expression information for 2024 genes, 115 GTs and 826 microRNAs was extracted from lung cancer, GT and microRNA expression databases, respectively.

We also conducted two different evaluations to measure the efficacy of DEXTER. The first evaluation focuses not only on the accuracy of Dexter but also the ability to ensure that the curation needs of BioXpress database are met, i.e. detection of differentially expressed genes in cancer tissues as compared with normal. In this evaluation, the system obtained a precision of 94% and recall of 84%. The second evaluation focused on general extraction of expression data in diseases from text and is not limited to BioXpress-specific sample comparison requirements. The system achieved precision and recall scores of 90 and 75%, respectively, in this evaluation.

## Materials and methods

In the subsequent sections, we will describe our approach to developing the DEXTER system. First we will introduce the types of expression information that are commonly found in the literature and discuss the ones we consider to be relevant for this task. Next, we formally describe the task and the different types of information that we extract. Finally, we present our system architecture and provide details of the extraction process.

### Types of expression information

Among the myriad statements in the literature regarding gene expression in disease, we have observed three broad categories:

Type A: In the first category are sentences that provide direct evidence of a gene’s expression in two differing scenarios, at least one of which involves a disease. These sentences are typically comparative; i.e. the expression of the gene is contrasted between the two scenarios (Example 1). Such sentences are common in the biomedical literature because experiments are frequently designed to compare two different samples or conditions. In a subset of these cases, the compared groups are cancerous and normal tissues; these are the sentences of interest to BioXpress.

Example 1: Expression of Shp2 protein was significantly upregulated in Oral Squamous Cell Carcinoma (OSCC) tissues compared with the normal tissues… .[PMID: 24439919]

Type B: In the second category are sentences that indicate the expression level of a gene in a disease state, but without an explicit comparison. In Example 2, expression of miR-155 is reported to be high in a disease sample (‘pancreatic cancer tissues’) without any explicit mention of a baseline.

Example 2: miR-155 expression was high in pancreatic cancer tissues. [PMID: 23817566]

Type C: In the third category are sentences that state the connection between a gene’s expression level and various disease-related concepts such as disease outcomes (e.g. ‘poor survival’) or disease processes (e.g. ‘metastasis’, ‘cancer cell proliferation’). Example 3 represents such a case. While such sentences are frequently found in the literature and inform us about the consequences of a gene’s expression, they do not address the association between the gene’s expression and the disease. For instance, from Example 3, we do not know whether C1GALT1 over-expression is typically observed in breast cancer; all we know is that *when* C1GALT1 is over-expressed in breast cancer, cell growth, migration and invasion are enhanced. Moreover, it is possible in these cases that the gene’s expression is being experimentally manipulated and is not a natural property of the disease cells at all. Therefore, we do not extract information from such sentences.

Example 3: Over-expression of C1GALT1 enhanced breast cancer cell growth, migration and invasion *in vitro* as well as tumor growth *in vivo*. [PMID: 25762620]

### Task definition

Based on the discussion above, we focused on information extraction from Type A and Type B sentences. For both types, DEXTER extracts the expressed gene/microRNA, the expression level, and the associated disease. For Type A sentences, where the expression is contrasted under two scenarios, it also extracts the compared scenarios. If one of the compared scenarios is normal tissues (e.g. Example 1), the results are flagged as being relevant to BioXpress. Information extracted from Type A sentences describing other compared scenarios (e.g. in Example 4, high vs. low grade tumors) and Type B sentences is also saved as it is of potential interest to researchers, clinicians and curators of other disease resources such as dbDEMC.

Example 4: expression levels of miR-454-3p were higher in high grade gliomas than in low grade gliomas. [PMID: 25190548]

To summarize, given a text, our tool, DEXTER, extracts:

- Expressed Gene/microRNA: the differentially expressed gene (normalized to NCBI Gene ID)/microRNA.
- Associated Disease: the disease associated with the sample where the gene is expressed. The disease is normalized to a Disease Ontology ID (26) (DOID).
- Expression Level: the level of expression, normalized to either ‘High’ or ‘Low’.
- Disease Sample: the sample (e.g. tissue, cell, cell line etc.) mentioned in the sentence, where the gene is expressed.
- Compared Sample: A second sample, which is used as a contrast to the sample in (d). This information is available in Type A, but not Type B, sentences.

Consider the sentence in Example 1. From this sentence we will extract the following:

- Shp2 (NCBI Gene ID: 5781), (b) OSCC (DOID: 0050866), (c) upregulated (High), (d) OSCC tissues and (e) normal tissues.

Note, that one of the motivations in developing DEXTER was to extend the literature-based portion of BioXpress. Since DEXTER can capture information in scenarios beyond BioXpress’s guidelines, we have to consider additional restrictions before DEXTER’s output can be integrated in BioXpress. For instance, the compared sample (e) is useful to determine inclusion in BioXpress, as BioXpress guidelines require comparison with normal or control samples. Thus, based on the information in (e), we determine that Example 1 will meet the guidelines of BioXpress, whereas Example 4 does not, since the compared sample is ‘normal tissues’ in Example 1 and ‘low grade gliomas’ in Example 4.

The disease sample (d) is the phrase used in text that mentions where the gene is expressed. For example, the disease sample is ‘OSCC tissues’ in Example 1 and ‘high grade gliomas’ in Example 4. As seen in these two examples, the disease sample (d) allows us to often infer the associated disease (b), which is normalized using DOID.

### System architecture

**Figure 1.**
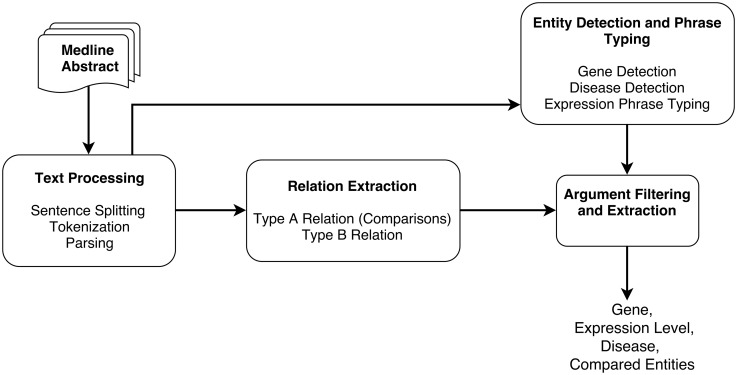
System pipeline overview.

The different steps of the DEXTER system are depicted in Figure 1. In the first step (text processing module in Figure 1), the title and text of a Medline abstract are split into sentences and tokenized. These sentences are then parsed to obtain syntactic dependencies between words and phrases. Since we treat this task as a relation extraction (RE) task, the RE module is applied, where two types of relations are extracted that correspond to Type A and Type B information. This RE phase relies on the syntactic dependencies identified in the previous step.

The output from the text processing component is also input to the entity detection and typing component. This component detects gene names, disease terms and phrases that mention expression information. Thus, the noun phrases (NPs) that have been identified by the parser can be checked to ascertain whether they are one of these entity types. After the RE step, we need to verify that the arguments are of the expected type. For example, the argument corresponding to compared scenario must be a disease or disease sample. Thus, we can filter out relations based on their argument’s type information. After filtering, additional processing may be necessary to extract some information. For instance, the disease information may not be stated in the sentence and might need to be extracted from elsewhere in the abstract. DEXTER is developed mainly using Python and Java programming languages. Details of each step of the system architecture are described in the subsequent subsections.

### Text processing

#### Preprocessing

In this preprocessing step, we tokenize and split the text, typically a Medline abstract, into sentences using the Stanford CoreNLP toolkit (27). After sentence splitting, we check whether a sentence contains certain words, the so-called trigger words for Type A and Type B relations (as described below). Sentences that do not contain trigger words are not processed further. A full list of trigger words that we use for expression and comparative relations can be found in the Supplementary File S1.

#### Syntactic processing

**Figure 2.**
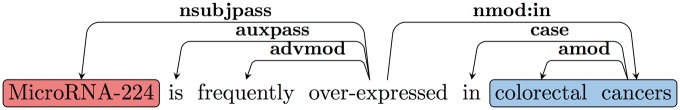
Example SDG.

The RE task is often defined as the identification of predicate-argument structures. We use a common approach to extract predicate–argument relations based on parsing. We further convert the syntactic parse tree into dependencies, in order to obtain an output that is closer to the predicate–argument relations. A standard dependency graph (SDG) (28) provides a representation of grammatical relations between words in a sentence. In Figure 2, an SDG using Universal Dependency notation for the sentence ‘MicroRNA-224 is frequently over-expressed in colorectal cancers’ is shown. One of the dependency triplets represented is nsubjpass (MicroRNA-224, over-expressed), where the relation is *nsubjpass* (nominal subject passive) and the governor and dependent of the relation are ‘over-expressed’ and ‘MicroRNA-224’, respectively.

We use the Charniak–Johnson parser (29, 30) with David McClosky’s adaptation to the biomedical domain (31) to obtain constituency parse trees for each sentence. Next, we use the Stanford conversion tool (27, 28) to convert the parse tree to into the syntactic dependency graph. We use the ‘CCProcessed’ option, which collapses and propagates dependencies allowing for an appropriate treatment of sentences that involve conjunctions. Note that ‘CCProcessed’ is helpful as dependencies involving preposition, conjuncts, as well as referent of relative clauses are ‘collapsed’ to get direct dependencies between context words.

Our approach to extracting information is based on defining patterns on the syntactic dependencies obtained after parsing. These patterns are written in Semgrex, which is a part of the Stanford NLP Toolkit. Semgrex allows us to specify the patterns as regular expressions based on lemmas, part-of-speech tags and dependency labels, which will automatically match with the dependency structure.

Text in biomedical literature is often complex and dense with information. While syntactic parsing provides an ability to abstract away from some textual variations, there are some syntactic variations that provide different dependency structures (active, passive and nominalized). However, these variations are systematic and have been captured in various linguistic theories as well as grammatical frameworks [e.g. tree families of lexicalized tree-adjoining grammars (LTAG)] (32, 33). In developing our patterns, we account for such syntactic variations, motivated by principles used in extended dependency graph (EDG) (34) and iXtractR (35).

### Relation extraction

As discussed earlier, we are interested in extracting information from sentences with and without explicit comparisons between groups (Type A and Type B, respectively). The following two subsections discuss the processing of the two different types of sentence.

#### Relations for Type A: comparison constructions

Recall that expression in disease samples for Type A information are present in comparative sentences, where the expression of a gene/microRNA is compared under two or more scenarios. The range of comparisons in biomedical literature is varied and extensive and obviously not limited to differential expression. In a previous work (36), we had developed a system that identifies and extracts information (components) from comparisons in general. While we refer to our earlier work for details of extraction of the comparison components, we briefly discuss the components of comparison here and how it relates to our task.

##### Components of comparison

Consider the sentence in Example 5, which compares a gene expression level in cancerous vs. non-cancerous tissues. Compared aspect (CA) is the aspect on which comparison between the two entities is being made. In this sentence, the CA is given by the phrase ‘The expression of GPC5 gene’. The compared scenarios will be referred to as compared entities (CEs) and are typically of the same type. In this example, the entities being compared are ‘lung cancer tissues’ and ‘adjacent noncancerous issues’. Additionally, there are two parts in comparative sentences that indicate the comparison. The first is the presence of a word that indicates the scale of the comparison and the other separates the two CEs. The former is often a comparative adjective or adverb (such as ‘higher’, ‘lower’, ‘better’ etc.), while the latter can be expressed with phrases or words (such as ‘than’, ‘compared with’, ‘versus’ etc.). We will refer to the comparative word indicating the scale as the scale indicator (SI) and the latter, separating the entities, as the entity separator (ES). In Example 5, these are given by ‘lower’ and ‘than’, respectively. Although the ES is useful for identifying the two entities and hence useful in our processing, we do not extract it as an argument and instead only extract the CA, the two CEs and the SI.

Example 5: The expression of GPC5 gene^CA^ was lower^SI^ in lung cancer tissues^CE^ than^ES^ in adjacent non-cancerous tissues^CE^.

Comparison sentences are written in a variety of textual and syntactic forms. Despite the variations, our previously developed method (36) effectively extracts the components of these comparisons by defining patterns based on syntactic dependencies, thereby abstracting away from the variations. Example 6 consists of seven sentences that illustrate some of the variety in comparative sentences in the literature; the components of the comparisons, extracted by our system, are shown in Table 1. In the first three sentences, the SI is the main predicate of the sentence. The SI in the first two sentences is a comparative adjective, whereas in the third sentence the SI is a verb. Regardless, in all three sentences the CA is the subject of the main predicate.

**Table 1.** Components extracted from Type A sentences (Example 6)

Sentence #	Scale indicator	Compared aspect	Compared entity 1	Compared entity 2
	(SI)	(CA)	(CE1)	(CE2)
1	Higher	Plasma miR-187	OSCC patients	Normal individuals
2	Lower	miR-181a expression	HepG2 cells	Hep3B cells
3	Higher	miR-210 expression	Metastatic tumors	Primary tumors
4	Increased	miR-95 levels	Human prostate cancer specimens	Normal tissues
5	Increased	TP expression	Ovarian cancers	Normal ovaries
6	Decreased	FOXD3 expression	HGG tissues	Normal brain tissues
7	Lower	Expression level of PTEN mRNA	Patients with CLL	Controls

In contrast, in the next two sentences (4 and 5), the SI is not the main predicate but is instead a noun modifier, modifying the CA. However, as with the first three sentences, the subject of the main predicate (and of the sentence) provides the CA.

Thus far, we have observed that the CA appears as the subject of the main predicate and that the SI acts as the main predicate of the sentence or is attached as a modifier of the CA NP. One of the CEs appears after the main predicate and is syntactically attached to the predicate via the use of the preposition ‘in’. The second CE is found later and separated by the ES.

The sixth and seventh sentences are different because the comparison structure starts with an ES phrase that includes the second CE. Other than this difference, the sixth sentence meets the conditions discussed above regarding the CA, the first CE and the SI. However, in the seventh sentence, one of the CE (‘patients with CLL’) is the subject of the main predicate. The CA (‘expression level of PTEN mRNA’) is the object of the predicate, with the SI (‘lower’) attached to the as a noun modifier.

All comparison patterns are listed in the Supplementary File S1.

Example 6:

1. Plasma miR-187 was significantly higher in OSCC patients than in normal individuals.
2. miR-181a expression was lower in HepG2 cells compared to Hep3B cells.
3. miR-95 levels were increased in human prostate cancer specimens compared with normal tissues.
4. Higher miR-210 expression was found in metastatic tumors compared to primary tumors.
5. Increased TP expression was observed in ovarian cancers than in normal ovaries.
6. In comparison to normal brain tissues, FOXD3 expression was significantly decreased in HGG tissues at both mRNA and protein levels.
7. Compared to controls, patients with CLL presented a lower expression level of PTEN mRNA (*P* < 0.001).

##### Extracting components from Type A (comparative) sentences

While we refer the reader to (36) for a full description of our system, we briefly describe the extraction process below and provide an example.

Recall that our approach to extracting components from comparative sentences is based on defining patterns, with Semgrex, on the syntactic dependencies obtained after parsing. We use dependency edges from SI and ES words to extract the CA and the CEs. An example of such a comparison pattern is described below.

**Figure 3.**
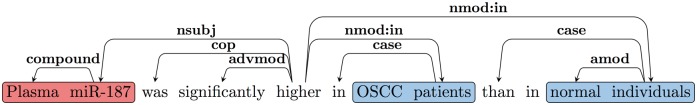
Comparison SDG example.

Figure 3 shows the dependency graph of a comparison sentence, where the SI (‘higher’), comparative adjective (JJR) serves as the main predicate of the sentence. Notice we normally expect the subject for the SI for such cases to be the CA. Thus, we follow the *nsubj* edge from the JJR (‘higher’) to get the head of the CA (‘miR-187’). We follow all outgoing edges from the CA head to extract the CA NP (‘Plasma miR-187’). As discussed earlier, one of the CE will be attached to the SI by the preposition ‘in’ in such cases. Thus, we use the *nmod: in* edges from JJR to extract the CEs (‘OSCC patients’ and ‘normal individuals’). We further verify that the extracted CEs are separated by an ES (‘than’).

#### Relation extraction for Type B

Type B sentences indicate the expression level of an entity (e.g. gene) in some disease sample, without explicitly contrasting it with another state. Importantly, an expression level for the entity, not just the entity itself, is mentioned. Hence we are interested in the (i) Expressed Aspect (EA): the entity being expressed, (ii) Expressed Location (EL): the biological context of the expressed entity, which can be disease samples, cells, tissues etc. and (iii) Level Indicator (LI): a phrase indicating the level of expression.

Several Type B sentences are shown in Example 7, depicting possible syntactic and textual variations. We show the output of our relations extraction system for these sentences in Table 2. In the first three sentences, the LI is the main predicate of the sentence, given by a verb, comparative adjective and adjective, respectively. Regardless of the type of LI, in these cases the subject of the main predicate provides us with the EA.

**Table 2.** Components extracted from Type B sentences (Example 7)

Sentence #	Level indicator	Expressed aspect	Expression location	Implicit comparison
	(LI)	(EA)	(EL)	
1	Over-expressed	GALNT2	OSCC	Yes
2	Higher	IGF1R expression levels	Right adrenocortical tumor	Yes
3	Low	Levels of miR-373 expression	Pancreatic cancer cell lines	No
4	Higher	Higher level of BRF2 expression	NSCLC tissues	Yes
5	High	TRIM32 expression levels	Gastric cancer tissues	No
6	High	Expression levels of FKBP51	Melanoma cells	No

In contrast, in the next two sentences (4 and 5), the main predicate of the sentence is headed by verbs such as ‘found’, ‘detected’, ‘noted’, ‘observed’ etc. In these cases, the LI is attached as a noun modifier, modifying the EA. But as with the first three sentences, the subject of the main predicate gives the EA. The EL in all these sentences (1–5) appears after the main predicate and is syntactically attached to the predicate via the preposition ‘in’. In the sixth sentence, the subject of the main predicate (‘found’) is ‘we’ and not the EA phrase. In such cases, where the subject are words such as ‘we’, ‘authors’, ‘study’ etc., the object of the predicate provides us with the EA and the EL is attached to the EA via the preposition ‘in’. As in seen sentences 4 and 5, the LI (‘high’) in this case is also attached to the EA as a noun modifier.

Example 7:

1. GALNT2 is frequently over-expressed in OSCC, especially in the carcinoma cells at the invasive front. [PMID: 24582885]
2. IGF1R expression levels were higher in the right adrenocortical tumor. [PMID: 21468523]
3. The levels of miR-373 expression were low in pancreatic cancer cell lines. [PMID: 24748127]
4. H igher level of BRF2 expression was found in NSCLC tissues. [PMID: 24523874]
5. High TRIM32 expression levels were detected in gastric cancer tissues. [PMID: 28521418]
6. We found high expression levels of FKBP51 in melanoma cells. [15571967]

Note that certain LI words and phrases, such as ‘over/under-expressed’, ‘increased’, ‘decreased’ and ‘elevated’ indicate implicit comparison to an unstated baseline. For example, in Sentence 7.1 the use of predicate ‘overexpressed’ does not make sense if the expression level (‘high’) is not in reference to some baseline. Thus, we note an ‘Implicit Comparison’ flag (last column in Table 2) in addition to EA, EL, LI arguments based on the type of the LI phrase. The relevance of this flag is further discussed in the section ‘Determining compared entity type’.

##### Extracting components from Type B sentences

**Figure 4.**
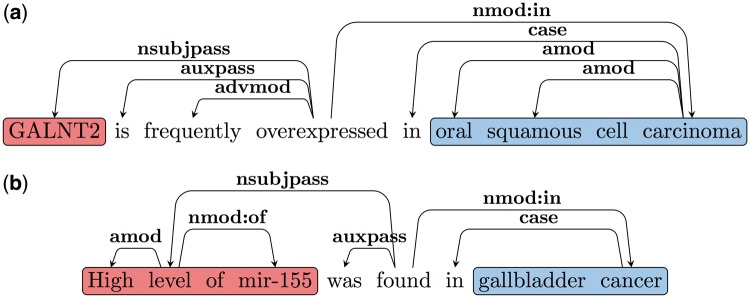
(a) Type B SDG Example 1. (b) Type B SDG Example 2.

Note that as discussed earlier there are two classes of predicates that trigger such Type B relations. The first class contains the LI, (e.g. ‘overexpressed in’, ‘under-expressed in’, ‘upregulated in’ and ‘increased in’). The second class includes words or multi-word triggers like: ‘is found in’, ‘is detected in’, ‘is increased in’ etc.; in these cases, the LI modifies the EA. Our patterns to extract the arguments of Type B relations are based on these two types of predicate classes. Consider the dependency graph shown in Figure 4a for Sentence 7.1. Here the LI is the main predicate of the sentence. Since the EA is the subject of the LI in these cases, we follow the *nsubjpass* edge from the predicate (LI) to obtain the EA (‘GALNT2’). We follow the *nmod: in* edge to extract the EL (‘oral squamous cell carcinoma’). An example dependency graph, where the LI is not the main predicate of the sentence is shown in Figure 4b. Here the main predicate is ‘found’ and similar to 4a *nsubj* and *nmod: in* edges are used to extract the EA and EL. The LI (‘high’) modifies the EA in these cases and hence we follow the *amod* edge from the head of the EA to obtain the LI. A complete list of patterns and triggers can be found in the Supplementary File S1.

### Entity detection and phrase typing

Since we are interested in extracting expression information in the context of disease, the arguments/components of our relations should satisfy certain type constraints. For example, in a comparison construction, the CA must of type *gene expression*. Further, our task requires the extraction of the expressed gene, expression level and the associated disease. Therefore, we need to determine the type of the argument phrases. In this phase, which takes parsed sentences as input from the text processing module, we look at NPs and determine if they contain terms that refer to entities of type *gene/miRNA, expression, or disease*/*disease-sample*.

Note that in this phase we only tag all the genes, microRNAs and diseases, expression and disease-sample phrases in text; details about how the particular expressed gene and the associated disease is extracted will be described in the section ‘Argument filtering and extraction’. Gene (we do not distinguish between genes and proteins in this phase) mentions are detected using PubTator (37), a publicly available tool that assists biocuration by tagging various biological entities. We downloaded and used the pre-computed annotations from PubTator, which contains gene mentions in abstracts normalized to NCBI Gene IDs. For microRNA detection, we use regular expressions that capture the ways in which they are mentioned in text (e.g. miR-1, microRNA1, miRNA-1 etc.). In developing the regular expressions, we have used the well-established naming convention as described in miRBase (38), including the detection of prefixes denoting species or suffixes as in miR-1a, miR-1-5p and hsa-miR-1-3p. To determine whether a phrase is of type ‘*Expression*’, we check the head noun of the phrase against a list of expression triggers such as ‘expression’, ‘level’, ‘over-expression’ etc. Note, the gene whose expression is defined by such an *Expression* phrase will either be in the same NP phrase indicating the *Expression* phrase or attached to it through a prepositional phrase. In both cases, the expressed gene will modify the Expression phrase such as in ‘X expression’ or ‘Expression of X’, where X is a name of a gene.

To detect diseases, we also use PubTator (37), where disease mentions are normalized to MEDIC IDs (39). These IDs are mapped to DOIDs using the table provided by Disease Ontology (DO) (26), which maps MEDIC IDs to DOIDs. The choice of normalizing diseases to DOIDs was made to allow easy integration to BioXpress, which only uses DOIDs. Note, that the arguments of our relation can be a disease (as detected by PubTator) or contain a disease with its head word matching certain *disease-sample* triggers such as ‘tissues’, ‘cells’, ‘patients’, ‘samples’, ‘tumors’ etc. A full list of expression and disease-sample triggers can be found in the Supplementary File S2.

### Argument filtering and extraction

There are two primary steps in this phase: (i) verify if the arguments found by the RE modules meet the type constraints and filter accordingly and (ii) extract the final relation that can be put into a database.

As shown in Figure 1, input to the RE module is a parsed sentence, which is also input to the entity and phrase typing module. A primary reason for this design is that we use a separately developed general purpose system for extracting comparisons. In this step, we check that the arguments from the RE module are of the right type, where the typing info has been determined by the typing module. Consider the comparison case: we verify that the phrase identified as the CA is of type *expression* and that the two CEs are of type *disease/disease-sample*. Similarly, we verify the type constraints for Type B: i.e. the EA is of type *gene* or *gene-expression* and the EL is of type *disease-sample*.

Next we discuss how to extract all the relevant information to populate a database. We first discuss the extraction of the gene and level and later discuss the extraction of the disease. The gene and level are always extracted from the sentence whereas the disease might be extracted from some other part of the abstract or its title.

#### Expressed gene and expression level extraction

Recall the CA and EA arguments of Type A and Type B are NP of type ‘*expression*’ or sometimes the gene itself. Thus these NPs will either directly contain the name of the gene we are capturing or the gene name will be attached to the *expression* phrase as a modifier. We use the gene/miRNA mentions detected in entity detection and typing module described in the section ‘Entity detection and phrase typing’ to extract the particular expressed gene/microRNA from the compared/EA arguments.

In addition to extracting the expressed gene, we also need to note the level of expression (high or low). As stated earlier the phrases, the expression level can be the predicate of the extracted relations (e.g. X *higher* in Y than Z, X *over-expressed* in Y) or attached to the compared/EA phrases as noun modifiers (e.g. *lower* expression of X was found in Y). These phrases are already captured by our RE system as SI or LI arguments for Type A and Type B relations, respectively. They are then normalized to high or low by matching them against a list of triggers. We use triggers such as ‘over-expressed’, ‘high’, ‘increased’ etc. to assign high expression level and triggers such as ‘under-expressed’, ‘low’, ‘decreased’ etc. to assign low. A full list of these triggers is listed in the Supplementary File S2.

#### Extracting the disease

In most cases, the disease is mentioned in the NPs corresponding to the CE or EL arguments of the Type A/B relations or attached to it by a prepositional phrase. Thus, while determining the associated disease, we check if a disease detected by PubTator (described in the section ‘Relation extraction’) is mentioned in one of the CEs or in the EL argument. In some cases, the arguments of the relations might only contain generic disease phrases such as ‘tumor’, ‘cancer’, ‘disease’ or population phrases such ‘patients’, ‘men’ etc. (as in the CEs in Example 6). In these cases, we assume that the referred disease can be inferred from context and the associated disease is extracted from elsewhere in the same abstract.

##### Inferring disease from context

Based on a preliminary study, we have noticed there are certain locations where the associated disease is stated. In some cases, title/first/conclusion sentences might contain the referred disease. These are locations where the authors tend to conclude or describe the nature of the work conducted. So any disease mentioned in these places are likely to be the disease studied in the presented work. For example, consider the sentence in Example 8. The CE argument extracted from this sentence is a generic disease phrase ‘cancer tissues’. The disease being referred to here is gastric cancer, which is mentioned in several places in the abstract including the title.

Example 8: Conversely, the expression of miR-143 and -195 in cancer tissues was significantly lower compared to that in normal tissues. [PMID: 24649051]

Alternately, the disease and the samples that were studied are described in the ‘methods’ part of the abstract, where descriptions of the investigational aims of the study or its setup are described. We have developed certain patterns to identify such sentences, which are described below.

Sentences that discuss the investigational aims of the paper contain certain *investigation* triggers, such as ‘investigated’, ‘examined’, ‘analyzed’, ‘evaluated’, ‘studied’, ‘compared’ etc. The presence of such triggers is not sufficient to detect such study/investigation sentences. We need to further verify that the investigation trigger has an appropriate agent. The agent (subject of the sentence) could be the authors of the paper, indicated by words, such as ‘we’ or ‘authors’ (Example 9a). Alternatively, the agent could be the reason for the investigation, indicated by words, such as ‘aim’, ‘objective’ or ‘purpose’ (Example 9b). Finally, the investigation trigger could be in the passive form with an optional agent as in Example 9c.

Example 9a: So the authors investigated the expression of TP in bladder cancer.

Example 9b: The purpose of this study was to investigate whether polyphenols from apples modulate expression of genes related to colon cancer prevention in preneoplastic cells derived from colon adenoma (LT97).

Example 9c: BACKGROUND: The association between 5-fluorouracil (5-FU)-related enzyme activity and the sensitivity of bladder urothelial carcinoma (BUC) to 5-FU were investigated, and methods to improve 5-FU sensitivity were analyzed.

Disease names can also be found in sentences describing the experimental set-up. These sentences contain certain *analyzed* trigger words such as ‘tested’, ‘enrolled’, ‘collected’, ‘analyzed’, ‘measured’, ‘explored’, ‘assessed’ etc. The patterns for detection of such sentences are similar to that of *investigation* sentences. The difference is that the theme of these words (i.e. who was enrolled/tested) will be patients or samples and will typically mention the disease being studied. We have noticed that most of the *analyzed* trigger constructs are in passive form and thus we look for the *nusbjpass* edge, which provides us with the theme (Examples 10a and 10b). The subject NP (underlined in the examples below) indicates the *sample* being tested/studied (theme) in the experiment. We look for a number, which often indicates how many patients/samples were tested, in the *sample* argument to further verify the detection of experiment setup sentences.

Example 10a: METHODS: A total of 140 patients with colorectal cancer and 280 cancer-free frequency-matched controls from a follow-up cohort population established in 1989, were enrolled.

Example 10b: Sera from 9 patients with chronic hepatitis B and 32 patients with hepatitis B virus (HBV)-related HCC were tested for AFP-L3 level using the glycan microarray.

#### Determining compared entity type

The CEs extracted from comparison constructions in Type A sentences should be a disease-sample such as disease cell, tissue, cell line, tumor, patients etc. Since BioXpress database guidelines require expression data that includes direct evidence of gene expression differences between tumor and adjacent non-tumor tissues (control), we differentiate between comparison to *Control* and *Not-Control* by adding a frame-of-reference flag. If one of CEs’ NP contain words such as ‘control’, ‘normal’, ‘healthy’, ‘adjacent’ etc. as a noun modifier, we detect the frame-of-reference as *Control* (Example 11) indicating the differential expression in disease vs. normal. If no such phrase is detected in the CEs, we set the flag to *Not-Control* as in Example 12, where the comparison is between two disease subtypes (‘T1 bladder carcinoma’ and ‘Ta carcinomas’).

Example 11: Higher TP expression was observed in ovarian cancers than in normal ovaries. [PMID: 15628771]

Example 12: ‘… .the expression of PDECGF in T1 bladder carcinoma was twofold higher than that in Ta carcinomas.’ [PMID: 9070497]

Note differential expression between tumor and normal can also be conveyed through certain predicate triggers of Type B relations such as ‘over/under-expressed’, ‘increased’, ‘decreased’, ‘elevated’ and ‘reduced’ (‘overexpressed’ as in Figure 4a). In addition to indicating high/low expression of the gene in cancer cells, these sentences also suggest an *implicit comparison to control*. The use of predicate ‘overexpressed’ used to detect high level of expression in the disease state does not make sense unless it is reference to some baseline. In such cases, we assume the comparison reference is normal (non-disease state) and the assign the frame-of-reference flag *Control_Implicit*. On the other hand, predicate triggers such as ‘high’, ‘low’ etc., indicates expression information but does not necessarily imply differential expression. In such cases, we assign *none* as the frame-of-reference as in Example 13.

Example 13: Expression of GCS was high in estrogen receptor (ER)-positive and HER-2 negative samples. [PMID: 24456584]

## Results

One of our motivations for designing DEXTER was to assist with curation of the BioXpress database. This section first discusses three use cases intended to extend the literature-based portion of the BioXpress database. In addition, we discuss the results of running DEXTER on a large set of PubMed abstracts related to cancer. Next we consider evaluation of DEXTER using standard measures of precision and recall. Our first evaluation focuses on results relevant to BioXpress and thus we only consider cases that compare gene expression in a cancer sample to a normal baseline. We also conducted a second evaluation in order to test DEXTER’s ability to extract expression data in diseases from text without the limitations imposed by BioXpress guidelines. Both evaluations are based on comparing DEXTER’s output with manually annotated data sets. The datasets were annotated by co-authors who are domain experts and did not participate in the design and implementation of the DEXTER system. The first evaluation used annotations by two researchers who are involved in the BioXpress database design. The second evaluation was based on annotations from a researcher who has considerable experience in biological curation and annotation.

### Use cases

Recall that one of the motivations for developing DEXTER was to extend the literature-based portion of the BioXpress database. DEXTER output is appropriate for BioXpress if: (i) the disease is cancer (as determined by DO) and (ii) there is an explicit/implicit comparison of expression in cancer samples to normal samples. The results of this section show that DEXTER can be scaled up to process and extract expression information from a large set of abstracts. We discuss the processing of three large sets of abstracts covering different use cases for inclusion in BioXpress. The processed text-mined results for these three sets have been integrated into the BioXpress database (https://hive.biochemistry.gwu.edu/bioxpress/about).

To address the use case in which a researcher wants to study a particular disease, we processed a set of abstracts related to lung cancer. Second, we focused on a set of abstracts related to a group of genes, namely GTs, which are a set of enzymes that play an important role in major post-translational modification in cellular development. Alteration to glycan structures or glycosylation status can play an important role in the development of neoplastic character in the proliferation cells. The last set of abstracts was selected to demonstrate that our method scales up to allow a comprehensive study, in this case for researchers interested in the role of microRNA’s in cancer. The substantial amount of data that we extracted for these three use case scenarios indicates that there is a wealth of information in the literature that DEXTER can extract. The selection methodology of the abstracts and some of the key characteristics of the three datasets developed are discussed below.

#### Use case 1: lung cancer

For our first set of abstracts, we focused on a specific cancer. Because of the OncoMX project (https://hive.biochemistry.gwu.edu/bioxpress; OncoMX website, which based on BioXpress is under development), which relies on BioXpress, we selected ‘lung cancer’ as the cancer of interest. We used DO to get all lung cancer terms, i.e. all terms in the DO hierarchy with lung cancer as root, which resulted in 47 DO cancer terms. We used synonyms provided for each lung cancer term by DO, resulting in a set of terms related to lung cancer. We queried PubMed with this list of terms (i.e. ‘lung cancer’ OR ‘lung carcinoma’ OR ‘non-small cell carcinoma’ …) to retrieve all lung cancer-related abstracts, which yielded 151, 618 abstracts. Next we selected only those abstracts that contain certain expression words/phrases such as ‘expression’, ‘level’ etc., which reduced the number of abstracts to 88 431 abstracts.

We ran DEXTER on these abstracts and selected only those results where the extracted cancer was one of the 47 lung cancer DOIDs. Table 3 (row 1) lists some of the key characteristics of lung cancer expression dataset. Note that the number of abstracts processed (88 431) by DEXTER reflects the number of abstracts that mentioned both lung cancer and the word expression (and a few more alternates) somewhere in the abstract. So in a large number of cases, the two were unrelated and might be several sentences apart. Even among the cases they appear in the same sentence, DEXTER is only concerned with mentions of differential expression in disease samples as compared to non-disease sample. We extracted Type A information from 742 abstracts and Type B information from 1448 abstracts. Total of 642 and 1383 genes were extracted from Type A and Type B expression information, respectively. Figure 5 depicts the top 10 genes extracted with the most literature evidence (number of abstracts, blue bars), in addition to the number of different lung cancer terms that were associated with each gene (orange bars). For example, differential expression for the top gene, epidermal growth factor receptor (EGFR) was extracted from 62 abstracts and was found be to expressed in ‘non-small lung carcinoma’, ‘lung cancer’, ‘lung oat cell carcinoma’, ‘lung small cell carcinoma’ and ‘lung adenocarcinoma’.

**Table 3.** Large-scale processing results

	# abstracts processed	# of abstracts extracted		# of entries		# of expressed genes	
		Type A	Type B	Type A	Type B	Type A	Type B
Lung cancer set	88 431	742	1 448	985	2019	642	1383
Glycosyltransferases set	27 516	90	180	106	236	42	73
microRNA set	28 067	1650	3575	2522	6437	477	721

**Figure 5.**
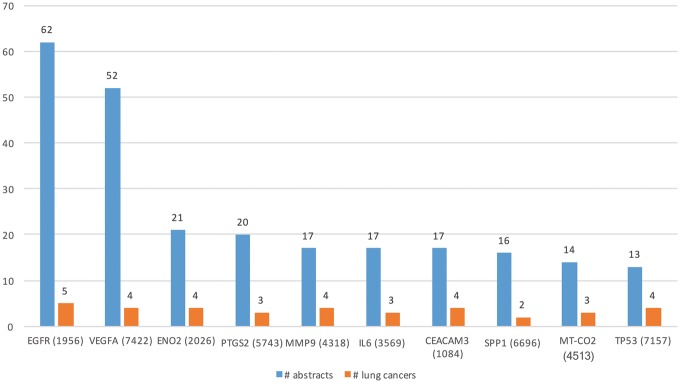
Top 10 genes whose expression is associated with lung cancer types in the literature.

#### Use Case 2: glycosyltransferases (GT) genes

For the second set of abstracts, we focused on expression information about a set of genes, GTs. GTs are responsible for attaching, extending, bifurcating and pruning glycans on proteins (40, 41). Dysfunction or deregulation of GTs may impact glycan profiles and lead to disease, including several types of cancer (42–44). For this project, GTs were defined as a set of 279 enzymes with one or several of the following: relevant annotations by gene ontology (GO) or UniProtKB/Swiss-Prot; GT classification by CAZY database; inclusion in the GT panel developed by the Consortium of Functional Glycomics; inclusion of appropriate domains reported by InterPro and Pfam.

We searched the precompiled PubTator gene database, which associates gene mentions in abstracts with their corresponding NCBI Gene IDs, for the 279 GTs and found 49, 915 relevant abstracts. As in the lung cancer set, we only selected those abstracts that contained expression words/phrases. Additionally, we only selected those abstracts that contained a cancer mention by selecting those abstracts that have a disease mention (as detected by PubTator) and whose MESH ID can be mapped any of the 2100 cancer DOID terms [cancer DOID terms are all nodes in the DO hierarchy with cancer (DOID:162) as root], using the DOID mapping file. These filtering steps yielded 27 516 abstracts on which we ran DEXTER and extracted BioXpress relevant information where the expressed gene was a GT.

**Figure 6.**
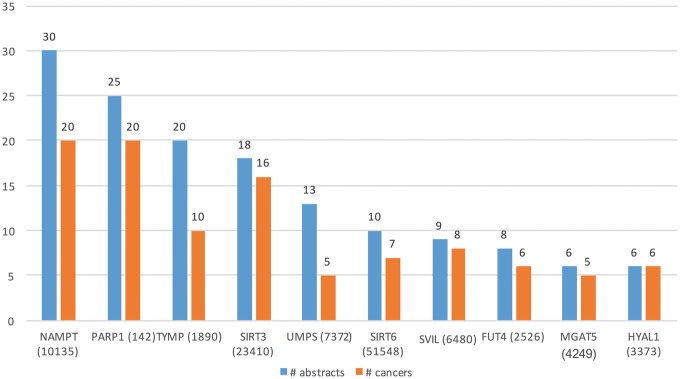
Top 10 GTs whose expression is associated with cancer types in the literature.

Table 3 (row 2) lists some of the key characteristics of GT expression dataset. From these 27 516 abstracts, we extracted Type A information from 90 abstracts and Type B from 180 abstracts. Total of 45 genes (in 34 cancers) and 73 (in 52 cancers) genes were extracted from Type A and Type B expression information, respectively. Figure 6 shows the top 10 GTs by number of abstracts (blue bars) and the number of cancer-related terms associated with them (orange bars). For example, differential expression for the top GTs, nicotinamide phosphoribosyltransferase (NAMPT) was extracted from 30 abstracts and was found be to expressed in 20 cancers, such as ‘stomach cancer’, ‘breast cancer’, ‘endometrial cancer’, ‘b-cell lymphoma’ and ‘papillary thyroid carcinoma’.

#### Use Case 3: microRNAs

For the third dataset, we processed all microRNA-related abstracts. We selected microRNAs as they transcriptionally regulate the expression of their target genes, and abnormalities in microRNA expression have been associated with many diseases. To select the microRNA abstracts, we used the PubMed query ‘microRNA[TIAB] OR miRNA[TIAB] OR miR[TIAB]’, which returned 64 995 abstracts. We followed the same filtering process as described in Use Case 2 for GTs genes, which reduced the number of abstracts to 28 067. We ran DEXTER on these abstracts and Table 3 (row 3) lists some of the key characteristics of microRNA expression dataset.

**Figure 7.**
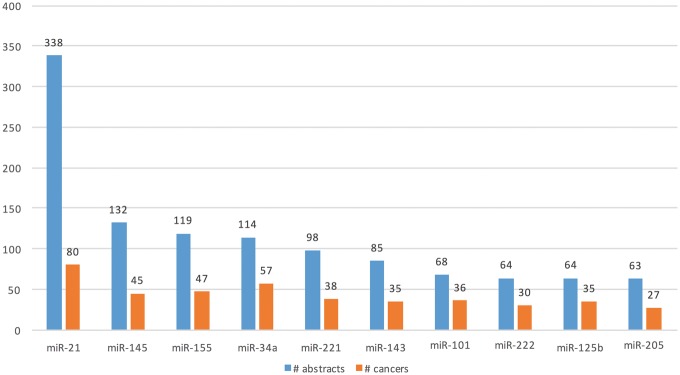
Top 10 microRNAs whose expression is associated with cancer types in the literature.

We extracted Type A information from 1 650 abstracts and Type B information from 3 575 abstracts. Total of 477 microRNAs (in 114 cancers) and 721 microRNAs (in 157 cancers) were extracted from Type A and Type B expression information, respectively. Figure 7 shows the top 10 extracted microRNAs with the most literature evidence (number of abstracts), and the number of cancers associated with each. For example, differential expression for the top microRNA, miR-21, was extracted from 338 abstracts and was found be to expressed in 80 different cancers, such as ‘breast cancer’, ‘hepatocellular carcinoma’, ‘colorectal cancer’, ‘stomach lymphoma’, ‘non-small cell lung carcinoma’ and ‘glioblastoma multiforme’.

### Large-scale processing

To illustrate the robustness and scalability of our tool, DEXTER was applied on a large set of PubMed abstracts related to cancer. To select cancer-related abstracts, we used the PubMed query ‘cancer OR cancers OR carcinoma OR carcinomas OR neoplasm OR neoplasms’, which returned 3 717 745 abstracts (as of March 2018). Next we selected only those abstracts that contain certain expression words/phrases, which reduced the number of abstracts to 1 750 928. We ran DEXTER on these abstracts and extracted differential expression information in cancer compared to normal, which resulted in 24 416 unique gene-cancer type pairs.

**Figure 8.**
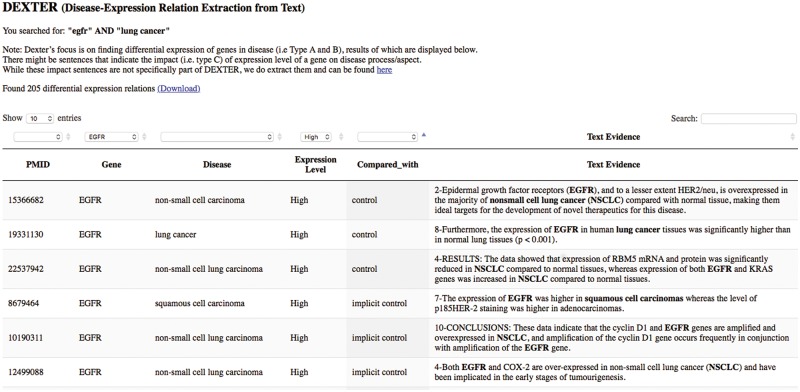
DEXTER web interface’s search results for the query ‘egfr AND lung cancer’.

We developed a preliminary website for interactive query of DEXTER’s text-mined results on cancer-related abstracts. The interface currently accepts PubMed-like queries as input, thus supporting queries like a gene name, or a disease name or any biological concept. For example, a user interested in the gene ‘egfr’ and the disease ‘lung cancer’ can submit a query such as ‘*egfr*’ *AND* ‘*lung cancer*’. The system then submits the query to PubMed, which returns all the PMIDs satisfying the query. The system displays the results from these PMIDs that have been previously processed by DEXTER. Because this list of PMIDs were returned by PubMed for the given query, the results may also contain results for genes other than ‘egfr’ and/or for other cancers. For this reason, the interface allows for filtering the results using drop-down menus. The interface is available at the URL: http://biotm.cis.udel.edu/DEXTER. Figure 8 provides a screenshot of the interface after submitting the query ‘egfr’ AND ‘lung cancer’.

Note that DEXTER’s focus is on extracting differential expression data in disease. But a clinical researcher could be interested in not only differential expression but also the impact (i.e. Type C) of expression level of a gene on disease aspect (e.g. ‘overall survival’) or process (e.g. ‘cell migration’). Even though Type C statements are outside the scope of DEXTER, we do extract them and have made them available for users of DEXTER. For this reason, for each search query in DEXTER’s interface, we also provide a download link to Type C statements that we collect for that query.

### BioXpress-based evaluation

#### Experimental setup

For this evaluation we selected 100 abstracts related to GTs and 100 abstracts related to microRNAs. To meet the needs of BioXpress, we need to extract gene/microRNA expression information where cancer is compared, explicitly or implicitly, with control. Therefore, our system identifies Type A sentences, where the one of the CEs has a modifier phrase suggesting it is a control sample (frame-of-reference flag *Control*) and Type B sentences, where there is an implicit comparison to control (frame-of-reference flag *Control_Implicit*). Since BioXpress is concerned only with expression information in the context of cancer and not other diseases, the abstracts in this evaluation set were selected if they contained some term likely to indicate cancer (e.g. tumor, malignant, cancer, carcinoma etc.).

To select the microRNA abstracts, we first used the PubMed query ‘microRNA[TIAB] OR miRNA[TIAB] OR miR[TIAB]’, which returned >60 000 abstracts. We further filtered and selected only those abstracts that mention a disease as detected by PubTator. Next we select only those abstracts, which contain certain expression words/phrases such as ‘expression’, ‘level’ etc., which reduces the number of abstracts to 28 067 abstracts. For selecting GT abstracts, instead of the using a PubMed query as with microRNAs, we identified the abstracts mentioning any of the GTs using the PubTator gene database. Then, as before, we selected a subset of abstracts that contained the expression words/phrases and a disease mention, which yielded 10 278 abstracts. Finally, we randomly selected 200 abstracts from these two sets with an equal number from each set.

Annotators marked the selected abstracts as relevant or not-relevant based on whether they met the criteria for inclusion in the BioXpress database. Only 90 of the 200 abstracts were annotated as relevant. When an abstract was annotated as relevant, the annotators also identified the associated disease, differentially expressed gene/microRNA and expression level.

#### Results

We ran DEXTER on the evaluation set and only considered output appropriate for BioXpress (i.e. differential expression comparing control and cancer samples). An instance was considered to be a true positive (TP) only when every individual component (expressed gene/microRNA, expression level and associated cancer) of DEXTER’s output matched the corresponding components in the annotation. Thus, an instance can be marked as false positive (FP) or false negative (FN) even if just one of the components (e.g. disease) of DEXTER’s output did not match the annotation. Table 4 shows the TP, FN, FP and precision (P), recall (R) and *F*-score (F) measures. The performance on the microRNA- and GT-related abstracts were almost the same.

**Table 4.** BioXpress-based evaluation results

True positive	False positive	False negative	Precision	Recall	*F*-score
77	5	15	93.90	83.69	88.51

### Second evaluation

#### Experimental setup

The first evaluation only considered cases where gene expression was compared between cancer and normal samples. Therefore, we conducted a second evaluation in order to test more general applications of our text-mining tool. We randomly selected 100 abstracts (divided equally among genes and microRNAs) as an evaluation set following the same procedure for abstract selection used in the first evaluation. This time the set of gene-related abstracts was not limited to GT genes but considered any gene. As before, the annotator marked all the expression information: expressed gene/microRNA, expression level and associated disease, which resulted in 169 annotated instances. In addition, if the annotator believed it was explicit comparison of expression level between two different scenarios, then the annotator also marked the two CEs.

#### Results

We ran DEXTER on the evaluation set and compared the output with the annotations. Similar to in the first evaluation, an instance was considered to be TP only when every component of DEXTER’s output, including the CEs matched corresponding component in the annotation. Table 5 shows the TP, FN, FP and P, R and *F* measures for the second evaluation.

**Table 5.** Second evaluation results

True positive	False positive	False negative	Precision	Recall	*F*-score
126	13	43	90.06	74.56	81.81

### Error analysis

We conducted an error analysis with the goal of improving our system. We noticed errors were due to mis-parsing, errors in disease detection, presence of anaphora or lack of patterns. An example of the last type is shown in Example 14. We were unable to capture this case as a Type A relation, because in this case the comparison spans two separate clauses. Because our current set of patterns relies entirely on parsing, it is not possible for the existing system to capture comparisons (hence Type A relations) where the two CEs appear in different clauses.

Example 14: Normal human colon cells express low levels of LEF1 and high levels of miR26b; however, human colon cancer cells have decreased miR26b expression and increased LEF1 expression. [PMID: 24785257]

Another type of error involved cases missed due to insufficient triggers. For example, consider the sentence (Example 15), which appears in an abstract used for the second evaluation. The implicit comparison in this sentence was missed by DEXTER because it does not use words like ‘after’ or ‘following’ as ES. Notice the comparison here is before and after an event, typically a treatment course. This example requires adding new triggers for ES, such as ‘after’, and ‘following’. Note that while the precision is roughly the same in both evaluations, the recall is lower in the second evaluation. We believe this might be due to the stricter guidelines adopted in the first evaluation and more errors encountered due to a greater variety of sentence structures in the second evaluation.

Example 15: Plasma concentrations of miR-208 increased significantly (*P* < 0.0001) after isoproterenol-induced myocardial injury and showed a similar time course to the concentration of cTnI, a classic biomarker of myocardial injury. [PMID: 19696117]

Another class of false negatives involved sentences where we were unable to infer the CA/entity from context (elsewhere in the abstract). These include cases where either the CA/CE is not mentioned in the sentence (as in Example 16a) or mentioned as anaphora and requires anaphora/reference resolution (as in Example 16b). For instance, in Example 16a, we failed to extract the Type A relation, since the CEs (‘PNI tumors’,

‘non-PNI-tumors’) were mentioned in a previous sentence. In Example 16b, we correctly extracted the Type A relation from the sentence, but were unable to extract the microRNAs (‘miR-192, miR-194, and miR-215’) being referred to in the CA argument (‘same microRNA’).

Example 16a: The most differently expressed microRNA was miR-224. [PMID: 18459106]

Example 16b: The same microRNAs were detected at high levels in normal colon tissue but were severely reduced in many colon cancer samples. [PMID: 19074875]

## Conclusion

In this paper, we have described DEXTER, a text-mining tool for extraction of gene and microRNA expression in disease samples. We have considered two types of sentences indicative of such expression information with (Type A) or without (Type B) an explicit comparison. From comparative (Type A) sentences we also extract the scenarios in which the expression of the gene/microRNA is contrasted (e.g. disease vs. control). This is particularly useful in capturing the classes of differential expression analyses relevant to the processes of neoplastic transformation and progression such as expression in cancer vs. respective normal tissue, high grade vs. low grade samples, metastasis vs. primary cancer etc. Our approach is based on RE, which relies on syntactic dependencies and general linguistic principles to handle different syntactic variations in text.

We have conducted two different evaluations to measure the efficacy of our text-mining tool. The first evaluation focused on differential gene/microRNA expression in cancer vs. normal samples; the second was more general, covering any description of differential gene/microRNA expression in the context of disease. The system achieved average *F*-scores of 88.51 and 81.81% for the first and second evaluation, respectively. We performed error analysis and the system will be improved to avoid such errors and other errors that we may find based on feedback from users. The tool currently works only on abstracts and not on full length articles. While the RE module might be applicable to full-text articles, since sometimes we might go beyond the current sentence to detect the disease, some changes might be required to extend this disease inference for full text. In the future, we plan to extend DEXTER to mine from full length articles.

DEXTER’s text-mined results can be used to streamline and accelerate curation of expression databases such as miR2Disease, dbDEMC. DEXTER’s results have already being integrated in BioXpress and in the future DEXTER’s extraction will be included directly in the literature-based portion of BioXpress as and when new sets are processed. These results can be obtained from the BioXpress download page. DEXTER’s text-mined will be tagged with a verified flag when a BioXpress curator manually verifies the output.

To show the scalability of DEXTER and the amount of information that can be extracted by DEXTER, we have also considered three use cases and processed all Medline abstracts for three use cases (lung cancer, 279 GTs genes and all microRNAs) and developed different expression databases containing direct evidence of gene expression differences between tumor and adjacent non-tumor tissues. In addition to access via BioXpress, DEXTER’s results (including these three use cases) are available at link mentioned below (http://biotm.cis.udel.edu/DEXTER). We anticipate that DEXTER results will also be of interest to researchers interested in a variety of questions relating to gene and microRNA expression in the context of disease.

## Supplementary data

Supplementary data are available at *Database* Online.

## Funding

Research reported in this manuscript is supported by the National Institutes of Health under Grants No. 1U01CA215010-01 and No. 5U01GM120953-02. The funders had no role in study design, data collection and analysis, decision to publish or preparation of the manuscript. Funding to pay the Open Access publication charges for this article was provided by the National Institutes of Health under Grant No. 1U01CA215010-01.

*Conflict of interest*. None declared.

## Supplementary Material

Supplementary Data 1

Supplementary Data 2

## References

[1] Lee T.I., Young R.A. (2013) Transcriptional regulation and its misregulation in disease. Cell, 152, 1237–1251. 23498934 10.1016/j.cell.2013.02.014 PMC3640494

[2] Fabian M.R., Sonenberg N., Filipowicz W. (2010) Regulation of mRNA translation and stability by microRNAs. Annu. Rev. Biochem., 79, 351–379. 20533884 10.1146/annurev-biochem-060308-103103

[3] Blenkiron C., Miska E.A. (2007) miRNAs in cancer: approaches, aetiology, diagnostics and therapy. Hum. Mol. Genet., 16, R106–R113. 17613543 10.1093/hmg/ddm056

[4] Greco S., Gorospe M., Martelli F. (2015) Noncoding RNA in age-related cardiovascular diseases. J. Mol. Cell. Cardiol., 83, 142–155. 25640162 10.1016/j.yjmcc.2015.01.011 PMC5509469

[5] Moura J., Børsheim E., Carvalho E. (2014) The role of microRNAs in diabetic complications-special emphasis on wound healing. Genes, 5, 926–956. 25268390 10.3390/genes5040926 PMC4276920

[6] Maciotta S., Meregalli M., Torrente Y. (2013) The involvement of microRNAs in neurodegenerative diseases. Front. Cell. Neurosci., 7, 265. 24391543 10.3389/fncel.2013.00265 PMC3867638

[7] Gori M., Arciello M., Balsano C. (2014) MicroRNAs in nonalcoholic fatty liver disease: novel biomarkers and prognostic tools during the transition from steatosis to hepatocarcinoma. Biomed. Res. Int., 2014, 1. 10.1155/2014/741465 PMC3972908 24745023

[8] Chapman C.G., Pekow J. (2015) The emerging role of miRNAs in inflammatory bowel disease: a review. Therap. Adv. Gastroenterol., 8, 4–22. 10.1177/1756283X14547360 PMC4265084 25553076

[9] Nalejska E., Mączyńska E., Lewandowska M.A. (2014) Prognostic and predictive biomarkers: tools in personalized oncology. Mol. Diagn. Ther., 18, 273–284. 24385403 10.1007/s40291-013-0077-9 PMC4031398

[10] Barrett T., Edgar R. (2006) Mining microarray data at NCBI’s Gene Expression Omnibus (GEO)*. Methods Mol. Biol., 338, 175–190. 16888359 10.1385/1-59745-097-9:175 PMC1619899

[11] Parkinson H., Sarkans U., Shojatalab M. et al (2004) ArrayExpress–a public repository for microarray gene expression data at the EBI. Nucleic Acids Res., 33, D553–D555. 10.1093/nar/gki056 PMC540010 15608260

[12] Zhang J., Baran J., Cros A. et al (2011) International Cancer Genome Consortium Data Portal—a one-stop shop for cancer genomics data. Database, 2011, bar026. 21930502 10.1093/database/bar026 PMC3263593

[13] The Cancer Genome Atlas Research Network, Weinstein J.N., Collisson E.A. et al (2013) The Cancer Genome Atlas Pan-Cancer analysis project. Nat. Genet., 45, 1113–1120. 24071849 10.1038/ng.2764 PMC3919969

[14] Liu X., Yu X., Zack D.J. et al (2008) TiGER: a database for tissue-specific gene expression and regulation. BMC Bioinformatics, 9, 271. 18541026 10.1186/1471-2105-9-271 PMC2438328

[15] Li H., He Y., Ding G. et al (2010) dbDEPC: a database of differentially expressed proteins in human cancers. Nucleic Acids Res., 38, D658–D664. 19900968 10.1093/nar/gkp933 PMC2808941

[16] He Y., Zhang M., Ju Y. et al (2012) dbDEPC 2.0: updated database of differentially expressed proteins in human cancers. Nucleic Acids Res., 40, D964–D971. 22096234 10.1093/nar/gkr936 PMC3245147

[17] Piñero J., Queralt-Rosinach N., Bravo À. et al (2015) DisGeNET: a discovery platform for the dynamical exploration of human diseases and their genes. Database, 2015, bav028. 25877637 10.1093/database/bav028 PMC4397996

[18] Bauer-Mehren A., Rautschka M., Sanz F. et al (2010) DisGeNET: a Cytoscape plugin to visualize, integrate, search and analyze gene–disease networks. Bioinformatics, 26, 2924–2926. 20861032 10.1093/bioinformatics/btq538

[19] Jiang Q., Wang Y., Hao Y. et al (2009) miR2Disease: a manually curated database for microRNA deregulation in human disease. Nucleic Acids Res., 37, D98–104. 18927107 10.1093/nar/gkn714 PMC2686559

[20] Wang D., Gu J., Wang T. et al (2014) OncomiRDB: a database for the experimentally verified oncogenic and tumor-suppressive microRNAs. Bioinformatics, 30, 2237–2238. 24651967 10.1093/bioinformatics/btu155

[21] Xie B., Ding Q., Han H. et al (2013) miRCancer: a microRNA-cancer association database constructed by text mining on literature. Bioinformatics, 29, 638–644. 23325619 10.1093/bioinformatics/btt014

[22] Wan Q., Dingerdissen H., Fan Y. et al (2015) BioXpress: an integrated RNA-seq-derived gene expression database for pan-cancer analysis. Database, 2015, 1–13. 10.1093/database/bav019 PMC4377087 25819073

[23] Dingerdissen H.M., Torcivia-Rodriguez J., Hu Y. et al (2017) BioMuta and BioXpress: mutation and expression knowledgebases for cancer biomarker discovery. Nucleic Acids Res., 46, D1128–D1136. 10.1093/nar/gkx907 PMC5753215 30053270

[24] Yang Z., Ren F., Liu C. et al (2010) dbDEMC: a database of differentially expressed miRNAs in human cancers. BMC Genomics, 11, S5. 10.1186/1471-2164-11-S4-S5 PMC3005935 21143814

[25] Yang Z., Wu L., Wang A. et al (2017) dbDEMC 2.0: updated database of differentially expressed miRNAs in human cancers. Nucleic Acids Res., 45, D812–D818. 27899556 10.1093/nar/gkw1079 PMC5210560

[26] Schriml L.M., Arze C., Nadendla S. et al (2012) Disease Ontology: a backbone for disease semantic integration. Nucleic Acids Res., 40, D940–D946. 22080554 10.1093/nar/gkr972 PMC3245088

[27] Manning C.D., Surdeanu M., Bauer J. et al (2014) The Stanford CoreNLP natural language processing toolkit. *Proceedings of 52nd Annual Meeting of the Association for Computational Linguistics: System Demonstrations*, ACL, Baltimore, MD, USA. pp. 55–60.

[28] De Marneffe M.-C., Dozat T., Silveira N. et al (2014) Universal Stanford dependencies: a cross-linguistic typology. *Proceedings of the Ninth International Conference on Language Resources and Evaluation*, European Language Resources Association, Reykjavík, Iceland. Vol. 14, pp. 4585–4592.

[29] Charniak E. (2000) A maximum-entropy-inspired parser. *Proceedings of the 1st North American Chapter of the Association for Computational Linguistics Conference, NAACL, 2000*. Association for Computational Linguistics, Stroudsburg, PA, USA. pp. 132–139.

[30] Charniak E., Johnson M. (2005) Coarse-to-fine n-best parsing and MaxEnt discriminative reranking. *Proceedings of the 43rd Annual Meeting on Association for Computational Linguistics, ACL, 2005*. Association for Computational Linguistics, Stroudsburg, PA, USA. pp. 173–180.

[31] Mcclosky D. (2010) Any Domain Parsing: Automatic Domain Adaptation for Natural Language Parsing. Brown University, Providence, RI.

[32] Schabes Y. (1992) Stochastic lexicalized tree-adjoining grammars. *Proceedings of the 14th Conference on Computational Linguistics - Volume 2, COLING, 1992*. Association for Computational Linguistics, Stroudsburg, PA, USA. pp. 425–432.

[33] Chen J., Shanker V.K. (2004) Text, speech and language technology. In: Bunt H., Carroll J., Satta G. (eds). New Developments in Parsing Technology. Springer, Netherlands, pp. 73–89.

[34] Peng Y., Gupta S., Wu C.H. et al (2015) An extended dependency graph for relation extraction in biomedical texts, *ACL-IJCNLP 2015*, p. 21.

[35] Peng Y., Torii M., Wu C.H. et al (2014) A generalizable NLP framework for fast development of pattern-based biomedical relation extraction systems. BMC Bioinformatics, 15, 285. 25149151 10.1186/1471-2105-15-285 PMC4262219

[36] Gupta S., Mahmood A.A., Ross K. et al (2017) Identifying comparative structures in biomedical text. BioNLP, 2017, 206–215.

[37] Wei C.-H., Kao H.-Y., Lu Z. (2013) PubTator: a web-based text mining tool for assisting biocuration. Nucleic Acids Res., 41, W518–W522. 23703206 10.1093/nar/gkt441 PMC3692066

[38] Griffiths-Jones S., Grocock R.J., van Dongen S. et al (2006) miRBase: microRNA sequences, targets and gene nomenclature. Nucleic Acids Res., 34, D140–D144. 16381832 10.1093/nar/gkj112 PMC1347474

[39] Davis A.P., Wiegers T.C., Rosenstein M.C. et al (2012) MEDIC: a practical disease vocabulary used at the Comparative Toxicogenomics Database. Database, 2012, bar065. 22434833 10.1093/database/bar065 PMC3308155

[40] Albesa-Jové D., Guerin M.E. (2016) The conformational plasticity of glycosyltransferases. Curr. Opin. Struct. Biol., 40, 23–32. 27450114 10.1016/j.sbi.2016.07.007

[41] Hossler P., Mulukutla B.C., Hu W.-S. (2007) Systems analysis of N-glycan processing in mammalian cells. PLoS One, 2, e713. 17684559 10.1371/journal.pone.0000713 PMC1933599

[42] Lopez-Sambrooks C., Shrimal S., Khodier C. et al (2016) Oligosaccharyltransferase inhibition induces senescence in RTK-driven tumor cells. Nat. Chem. Biol., 12, 1023–1030. 27694802 10.1038/nchembio.2194 PMC5393272

[43] Ono M., Tsuda H., Kobayashi T. et al (2015) The expression and clinical significance of ribophorin II (RPN2) in human breast cancer. Pathol. Int., 65, 301–308. 25881688 10.1111/pin.12297

[44] Dong S., Wang Z., Huang B. et al (2017) Bioinformatics insight into glycosyltransferase gene expression in gastric cancer: pOFUT1 is a potential biomarker. Biochem. Biophys. Res. Commun., 483, 171–177. 28040433 10.1016/j.bbrc.2016.12.172

