# Supplementary material for: DEXTER: Disease-Expression Relation Extraction from Text
Source: Database (Oxford). 2018 May 30;2018:bay045. doi: 10.1093/database/bay045 (PMC6007211; doi:10.1093/database/bay045)
Supplement: Supplementary Data 1 [file bay045_supp_s1.docx]

**SUPPLEMENTARY FILE S1**

####README START#####

The rule for adding new edges to comparison components consists of set of conditions and associated actions.

If all the conditions are satisfied (on the Standard Dependency Graph), then a set of nodes are populated.

Based on the populated nodes, user can write actions specifying between which nodes he/she wants to add edges.

The set of conditions are based on Stanford Semgrex pattern.

The sets of conditions (semregex patterns) are applied to the dependency and it populates a set of nodes based on the pattern.

Then the actions are applied to add new edges between the named nodes.

Each Cond_# is Semregex Pattern

Each Action_# adds edges to comparison(Type A)/TypeB components

####README END#####

**TYPE A COMPARSION PATTERNS**

####arg0_cmp points to the compared aspect

####arg1/2_cmp points to the compared entities

####governor of arg edges is the comparison scale

##########X higher/lower in Y CMP Z###############

####Comparison 1: X higher/lower in Y than in Z; X in Y higher/lower than in Z####

RuleID : cmp1_than_1

Cond_1 : {word:/(higher|lower|high|low)/}=N0

Cond_2 : {}=N0 >nsubj {}=N1

Cond_3 : [{}=N0 | {}=N1] >/nmod:in/ {}=N2

Cond_4 : {}=N2 !> /case/ {word:than}

Cond_5 : {}=N0 >/nmod:in/ ({}=N3 > /case/ {word:than})

Action_1 : N0 >> arg0_cmp >> N1

Action_2 : N0 >> arg1_cmp >> N2

Action_3 : N0 >> arg2_cmp >> N3

####Comparison 1_1: X higher/lower in Y versus Z; X in Y higher/lower versus Z####

RuleID : cmp1_vs_1

Cond_1 : {word:/(higher|lower|high|low)/}=N0

Cond_2 : {}=N0 >nsubj {}=N1

Cond_3 : [{}=N0 | {}=N1] >/nmod:in/ {}=N2

Cond_4 : {}=N2 !> /cc/ {word:/(versus|vs.)/}

Cond_5 : {}=N0 >/nmod:in/ ({}=N3 > /cc/ {word:/(versus|vs.)/})

Action_1 : N0 >> arg0_cmp >> N1

Action_2 : N0 >> arg1_cmp >> N3

Action_3 : N0 >> arg2_cmp >> N2

####Comparison 2: X higher/lower in Y than Z; X in Y higher/lower than Z####

RuleID : cmp1_than_2

Cond_1 : {word:/(higher|lower|high|low)/}=N0

Cond_2 : {}=N0 >nsubj {}=N1

Cond_3 : [{}=N0 | {}=N1] >/nmod:in/ {}=N2

Cond_4 : {}=N0 >/(nmod:than|nmod:than_added|conj:vs.|conj:versus)/ {}=N3

Action_1 : N0 >> arg0_cmp >> N1

Action_2 : N0 >> arg1_cmp >> N2

Action_3 : N0 >> arg2_cmp >> N3

####Comparison 3: X higher/lower in Y compared with|to Z; X in Y ..####

RuleID : cmp1_compare_1

Cond_1 : {word:/(higher|lower|high|low)/}=N0

Cond_2 : {}=N0 >nsubj {}=N1

Cond_3 : [{}=N0 | {}=N1] >/nmod:in/ {}=N2

Cond_4 : [{}=N0 | {}=N2] >/(advcl|nmod):(compared_to|compared_with)/ {}=N3

Action_1 : N0 >> arg0_cmp >> N1

Action_2 : N0 >> arg1_cmp >> N2

Action_3 : N0 >> arg2_cmp >> N3

####Comparison 4: X higher/lower in Y compared with|to in Z; X in Y ..####

RuleID : cmp1_compare_2

Cond_1 : {word:/(higher|lower|high|low)/}=N0

Cond_2 : {}=N0 >nsubj {}=N1

Cond_3 : [{}=N0 | {}=N1] >/nmod:in/ {}=N2

Cond_4 : {}=N0 > /advcl:compared/ {word:/(to|with)/}

Cond_9 : {}=N2 $++ {}=N3

Cond_10 : [{}=N0 | {}=N1] >/nmod:in/ {}=N3

Action_1 : N0 >> arg0_cmp >> N1

Action_2 : N0 >> arg1_cmp >> N2

Action_2 : N0 >> arg2_cmp >> N3

####Comparison 5: X higher/lower in Y when compared with|to Z; X in Y ..####

RuleID : cmp1_compare_3

Cond_1 : {word:/(higher|lower|high|low)/}=N0

Cond_2 : {}=N0 >nsubj {}=N1

Cond_3 : [{}=N0 | {}=N1] >/nmod:in/ !{word:/(compared|comparison)/}=N2

Cond_4 : {}=N0 >/(advcl|dep|nmod:in|nmod_acl:in)/ {word:/(compared|comparison)/}=N4

Cond_5 : {}=N4 >/(nmod:in|nmod:with|nmod:to)/ {}=N3

Action_1 : N0 >> arg0_cmp >> N1

Action_2 : N0 >> arg1_cmp >> N2

Action_3 : N0 >> arg2_cmp >> N3

########X [DIFF_EXPRESSED_IN] Y CMP Z##############

####Comparison 1': X increased in Y than in Z; X in Y increased than in Z####

RuleID : cmp2_than_1

Cond_1 : {pos:VBN;lemma:/.*(increase|decrease|express|silence|reduce|elevate|change|regulate)/}=N0

Cond_2 : {}=N0 >nsubjpass {}=N1

Cond_3 : [{}=N0 | {}=N1] >/nmod:in/ {}=N2

Cond_4 : {}=N2 !> /case/ {word:than}

Cond_5 : {}=N0 >/nmod:in/ ({}=N3 > /case/ {word:than})

Action_1 : N0 >> arg0_cmp >> N1

Action_2 : N0 >> arg1_cmp >> N2

Action_3 : N0 >> arg2_cmp >> N3

####Comparison 1'_1: X increased in Y versus Z; X in Y increased versus Z####

RuleID : cmp2_vs_1

Cond_1 : {pos:VBN;lemma:/.*(increase|decrease|express|silence|reduce|elevate|change|regulate)/}=N0

Cond_2 : {}=N0 >nsubjpass {}=N1

Cond_3 : [{}=N0 | {}=N1] >/nmod:in/ {}=N2

Cond_4 : {}=N2 !> /cc/ {word:/(versus|vs.)/}

Cond_5 : {}=N0 >/nmod:in/ ({}=N3 > /cc/ {word:/(versus|vs.)/})

Action_1 : N0 >> arg0_cmp >> N1

Action_2 : N0 >> arg1_cmp >> N3

Action_3 : N0 >> arg2_cmp >> N2

####Comparison 2': X increased in Y than Z; X in Y increased than Z####

RuleID : cmp2_than_2

Cond_1 : {pos:VBN;lemma:/.*(increase|decrease|express|silence|reduce|elevate|change|regulate)/}=N0

Cond_2 : {}=N0 >nsubjpass {}=N1

Cond_3 : [{}=N0 | {}=N1] >/nmod:in/ {}=N2

Cond_4 : {}=N0 >/(nmod:than|nmod:than_added|conj:vs.|conj:versus)/ {}=N3

Action_1 : N0 >> arg0_cmp >> N1

Action_2 : N0 >> arg1_cmp >> N2

Action_3 : N0 >> arg2_cmp >> N3

####Comparison 3': X increased in Y compared with|to Z; X in Y ..####

RuleID : cmp2_compare_1

Cond_1 : {pos:VBN;lemma:/.*(increase|decrease|express|silence|reduce|elevate|change|regulate)/}=N0

Cond_2 : {}=N0 >nsubjpass {}=N1

Cond_3 : [{}=N0 | {}=N1] >/nmod:in/ {}=N2

Cond_4 : [{}=N0 | {}=N2] >/(advcl|nmod):(compared_to|compared_with)/ {}=N3

Action_1 : N0 >> arg0_cmp >> N1

Action_2 : N0 >> arg1_cmp >> N2

Action_3 : N0 >> arg2_cmp >> N3

####Comparison 4': X increased in Y compared with|to in Z; X in Y ..####

RuleID : cmp2_compare_2

Cond_1 : {pos:VBN;lemma:/.*(increase|decrease|express|silence|reduce|elevate|change|regulate)/}=N0

Cond_2 : {}=N0 >nsubjpass {}=N1

Cond_3 : [{}=N0 | {}=N1] >/nmod:in/ {}=N2

Cond_4 : {}=N0 > /advcl:compared/ {word:/(to|with)/}

Cond_9 : {}=N2 $++ {}=N3

Cond_10 : [{}=N0 | {}=N1] >/nmod:in/ {}=N3

Action_1 : N0 >> arg0_cmp >> N1

Action_2 : N0 >> arg1_cmp >> N2

Action_2 : N0 >> arg2_cmp >> N3

####Comparison 5': X increased in Y when compared with|to Z; X in Y ..; X increased in Y in cmp to Z####

RuleID : cmp2_compare_3

Cond_1 : {pos:VBN;lemma:/.*(increase|decrease|express|silence|reduce|elevate|change|regulate)/}=N0

Cond_2 : {}=N0 >nsubjpass {}=N1

Cond_3 : [{}=N0 | {}=N1] >/nmod:in/ !{word:/(compared|comparison)/}=N2

Cond_4 : {}=N0 >/(advcl|dep|nmod:in|nmod_acl:in)/ {word:/(compared|comparison)/}=N4

Cond_5 : {}=N4 >/(nmod:in|nmod:with|nmod:to)/ {}=N3

Action_1 : N0 >> arg0_cmp >> N1

Action_2 : N0 >> arg1_cmp >> N2

Action_3 : N0 >> arg2_cmp >> N3

###########Higher/Lower of X [FOUND IN] Y CMP Z

####Comparison 1'': Higher of X found in Y than in Z; Higher of X in Y found than in Z####

RuleID : cmp3_than_1

Cond_1 : {word:/(found|noted|detected|observed|discovered|occurred|occur|occurs)/}=N0

Cond_2 : {}=N0 >/(nsubjpass|dobj)/ {}=N1

Cond_3 : [{}=N0 | {}=N1] >/nmod:in/ {}=N2

Cond_4 : {}=N2 !> /case/ {word:than}

Cond_5 : [{}=N0 | {}=N2] >/nmod:in/ ({}=N3 > /case/ {word:than})

Cond_6 : {}=N1 >amod {tag:/(JJ|JJR|VBN)/}=N4

Action_1 : N4 >> arg0_cmp >> N1

Action_2 : N4 >> arg1_cmp >> N2

Action_3 : N4 >> arg2_cmp >> N3

####Comparison 2'': Higher of X found in Y than Z; Higher of X in Y found than Z####

RuleID : cmp3_than_2

Cond_1 : {word:/(found|noted|detected|observed|discovered|occurred|occur|occurs)/}=N0

Cond_2 : {}=N0 >/(nsubjpass|dobj)/ {}=N1

Cond_3 : [{}=N0 | {}=N1] >/nmod:in/ {}=N2

Cond_4 : [{}=N0 | {}=N2] >/nmod:than|conj:vs.|conj:versus/ {}=N3

Cond_5 : {}=N1 >amod {tag:/(JJ|JJR|VBN)/}=N4

Action_1 : N4 >> arg0_cmp >> N1

Action_2 : N4 >> arg1_cmp >> N2

Action_3 : N4 >> arg2_cmp >> N3

####Comparison 2''_1: Higher of X found in Y versus Z; Higher of X in Y found versus Z####

RuleID : cmp3_vs_1

Cond_1 : {word:/(found|noted|detected|observed|discovered|occurred|occur|occurs)/}=N0

Cond_2 : {}=N0 >/(nsubjpass|dobj)/ {}=N1

Cond_3 : [{}=N0 | {}=N1] >/nmod:in/ {}=N2

Cond_4 : {}=N2 !> /cc/ {word:/(versus|vs.)/}

Cond_5 : {}=N0 >/nmod:in/ ({}=N3 > /cc/ {word:/(versus|vs.)/})

Cond_6 : {}=N1 >amod {tag:/(JJ|JJR|VBN)/}=N4

Action_1 : N4 >> arg0_cmp >> N1

Action_2 : N4 >> arg1_cmp >> N3

Action_3 : N4 >> arg2_cmp >> N2

####Comparison 3'': Higher of X found in Y compared with|to Z; X in Y ..####

RuleID : cmp3_compare_1

Cond_1 : {word:/(found|noted|detected|observed|discovered|occurred|occur|occurs)/}=N0

Cond_2 : {}=N0 >/(nsubjpass|dobj)/ {}=N1

Cond_3 : [{}=N0 | {}=N1] >/nmod:in/ {}=N2

Cond_4 : [{}=N0 | {}=N2] >/(advcl|nmod):(compared_to|compared_with)/ {}=N3

Cond_5 : {}=N1 >amod {tag:/(JJ|JJR|VBN)/}=N4

Action_1 : N4 >> arg0_cmp >> N1

Action_2 : N4 >> arg1_cmp >> N2

Action_3 : N4 >> arg2_cmp >> N3

####Comparison 4'': Higher of X found in Y compared with|to in Z; X in Y ..####

RuleID : cmp3_compare_2

Cond_1 : {word:/(found|noted|detected|observed|discovered|occurred|occur|occurs)/}=N0

Cond_2 : {}=N0 >/(nsubjpass|dobj)/ {}=N1

Cond_3 : [{}=N0 | {}=N1] >/nmod:in/ {}=N2

Cond_4 : {}=N0 > /advcl:compared/ {word:/(to|with)/}

Cond_5 : {}=N1 >amod {tag:/(JJ|JJR|VBN)/}=N3

Cond_9 : {}=N2 $++ {}=N4

Cond_10 : [{}=N0 | {}=N1] >/nmod:in/ {}=N4

Action_1 : N3 >> arg0_cmp >> N1

Action_2 : N3 >> arg1_cmp >> N2

Action_2 : N3 >> arg2_cmp >> N4

####Comparison 5'': Higher of X found in Y when compared with|to Z; X in Y ..; Higher of X found in Y in cmp to Z####

RuleID : cmp3_compare_3

Cond_1 : {word:/(found|noted|detected|observed|discovered|occurred|occur|occurs)/}=N0

Cond_2 : {}=N0 >/(nsubjpass|dobj)/ {}=N1

Cond_3 : [{}=N0 | {}=N1] >/nmod:in/ !{word:/(compared|comparison)/}=N2

Cond_4 : {}=N0 >/(advcl|dep|nmod:in|nmod_acl:in)/ {word:/(compared|comparison)/}=N4

Cond_5 : {}=N4 >/(nmod:in|nmod:with|nmod:to)/ {}=N3

Cond_5 : {}=N1 >amod {tag:/(JJ|JJR|VBN)/}=N5

Action_1 : N5 >> arg0_cmp >> N1

Action_2 : N5 >> arg1_cmp >> N2

Action_3 : N5 >> arg2_cmp >> N3

####Comparison 2.1: Y VB.* JJR X than/compared Z

RuleID : cmp_VBD_JJR_1

Cond_1 : {tag:/VBD/}=N0

Cond_2 : {}=N0 >nsubj {}=N1

Cond_3 : {}=N0 >/dobj/ {}=N2

Cond_4 : {}=N2 >amod {tag:JJR}=N3

Cond_5 : [{}=N2 | {}=N0 | {}=N1] >/nmod:than|(advcl|acl|nmod):compared_(to|with)/ {}=N4

Action_1 : N3 >> arg0_cmp >> N2

Action_2 : N3 >> arg1_cmp >> N1

Action_3 : N3 >> arg2_cmp >> N4

####arg_cmp that/those propagation####

RuleID : comparisonPropagation1

Cond_1 : {}=N0 >/arg.*_cmp/=R1 {word:/(that|those)/}=N1

Cond_2 : {}=N1 >/nmod:in/ {}=N2

Action_1 : N0 >> R1_prop >> N2

**TYPE B PATTERNS**

####arg0_exp or arg0_fnd points to the expressed aspect

####arg1_exp or arg0_exp points to the expressed location

####X is expressed in Y#####

RuleID : expressionIn_1

Cond_1 : {pos:VBN;lemma:/.*(increase|decrease|express|silence|reduce|elevate|change|regulate)/}=N0

Cond_2 : {}=N0 >/(nsubjpass|nsubj_null)/ {}=N1

Cond_3 : {}=N0 >/nmod:in/ {}=N2

Cond_10 : {}=N0 !>/.*_cmp/ {}

Action_1 : N0 >> arg0_exp >> N1

Action_2 : N0 >> arg1_exp >> N2

####X is expressed in Y#####

RuleID : expressionIn_2

Cond_1 : {pos:/(JJ|JJR)/;lemma:/(high|low|higher|lower)/}=N0

Cond_2 : {}=N0 >/(nsubj|nsubj_null)/ {}=N1

Cond_3 : {}=N0 >/nmod:in/ {}=N2

Cond_10 : {}=N0 !>/.*_cmp/ {}

Action_1 : N0 >> arg0_exp >> N1

Action_2 : N0 >> arg1_exp >> N2

####X is found in Y#####

RuleID : foundIn_1

Cond_1 : {word:/(found|noted|detected|observed|discovered|occurred|occur|occurs)/}=N0

Cond_2 : {}=N0 >/(nsubjpass|nsubj|nsubj_null)/ {}=N1

Cond_3 : {}=N0 >/nmod:in/ {}=N2

Cond_10 : {}=N0 !>/.*_cmp/ {}

Action_1 : N0 >> arg0_fnd >> N1

Action_2 : N0 >> arg1_fnd >> N2

####X in Y was found#####

RuleID : foundIn_2

Cond_1 : {word:/(found|noted|detected|observed|discovered|occurred|occur|occurs)/}=N0

Cond_2 : {}=N0 >/(nsubjpass|nsubj_null)/ {}=N1

Cond_3 : {}=N1 >/nmod:in/ {}=N2

Cond_10 : {}=N0 !>/.*_cmp/ {}

Action_1 : N0 >> arg0_fnd >> N1

Action_2 : N0 >> arg1_fnd >> N2

####We found X in Y#####

RuleID : foundIn_3

Cond_1 : {word:/(found|noted|detected|observed|discovered|occurred|occur|occurs)/}=N0

Cond_2 : {}=N0 >/dobj/ {}=N1

Cond_3 : {}=N1 >/nmod:in/ {}=N2

Cond_10 : {}=N0 !>/.*_cmp/ {}

Action_1 : N0 >> arg0_fnd >> N1

Action_2 : N0 >> arg1_fnd >> N2

####High X is found in Y#####

RuleID : EXPfoundIn_1

Cond_1 : {word:/(found|noted|detected|observed|discovered|occurred|occur|occurs)/}=N0

Cond_2 : {}=N0 >/(nsubjpass|nsubj|nsubj_null)/ {}=N1

Cond_3 : {}=N0 >/nmod:in/ {}=N2

Cond_4 : {}=N1 >amod {tag:/(JJ|JJR|VBN)/}=N3

Cond_10 : {}=N3 !>/.*_cmp/ {}

Action_1 : N3 >> arg0_exp >> N1

Action_2 : N3 >> arg1_exp >> N2

####High X in Y was found#####

RuleID : EXPfoundIn_2

Cond_1 : {word:/(found|noted|detected|observed|discovered|occurred|occur|occurs)/}=N0

Cond_2 : {}=N0 >/(nsubjpass|nsubj_null)/ {}=N1

Cond_3 : {}=N1 >/nmod:in/ {}=N2

Cond_4 : {}=N1 >amod {tag:/(JJ|JJR|VBN)/}=N3

Cond_10 : {}=N3 !>/.*_cmp/ {}

Action_1 : N3 >> arg0_exp >> N1

Action_2 : N3 >> arg1_exp >> N2

####We found high X in Y#####

RuleID : EXPfoundIn_3

Cond_1 : {word:/(found|noted|detected|observed|discovered|occurred|occur|occurs)/}=N0

Cond_2 : {}=N0 >/dobj/ {}=N1

Cond_3 : {}=N1 >/nmod:in/ {}=N2

Cond_4 : {}=N1 >amod {tag:/(JJ|JJR|VBN)/}=N3

Cond_10 : {}=N3 !>/.*_cmp/ {}

Action_1 : N3 >> arg0_exp >> N1

Action_2 : N3 >> arg1_exp >> N2
