# Supplementary material for: DEXTER: Disease-Expression Relation Extraction from Text
Source: Database (Oxford). 2018 May 30;2018:bay045. doi: 10.1093/database/bay045 (PMC6007211; doi:10.1093/database/bay045)
Supplement: Supplementary Data 2 [file bay045_supp_s2.docx]

**SUPPLEMENTARY FILE S2**

1. **Expression Phrase Typing Triggers**

“over-expression”, “under-expression”, “expression”, “up-regulation”, “down-regulation”,

“overexpression”, “underexpression”, “upregulation”, “downregulation”, “level”, “knockdown”, “elevation”, “production”, “silencing”, “loss”, “gain”, “depletion”, “absence”, “abundance”, “concentration”

1. Disease Sample Phrase Typing Triggers

“tissue”, cell”, “patient”, “sample”, “tumor”, “cancer”, “carcinoma”, “cell line”, “cell-line”, “group”, “blood”, “sera”, “serum”, fluid”, “subset”, “case”, “men”, “women”

NOTE: All valid plural forms are also considered

1. **Control Sample Phrase Typing Triggers**

control, normal, health, healthy, NC, adjacent, peri-tumoral, peritumoral,

non( |- )?(tumor|tumoral|cancerous)

NOTE: All valid plural forms are also considered

1. Expression Level Triggers

NOTE: All valid textual variations (tense and nominalized forms) of the triggers below are considered. For example, for the high expression level trigger “overexpressed” its nominal form “over-expression” is also considered.

A. High Expression Level triggers:

“gain”, “increased”, “high”, “overexpressed”, “over-expressed”, “positive”, “strong”, elevated”, “upregulated”, “up-regulated”, “higher”

B. Low Expression Level Triggers:

“loss”, “decreased”, “low”, “underexpressed”, “under-expressed”, ““down-regulated” “downregulated”, reduced”, “knockdown”, “suppressed”, “negative”, “weak”, “lower”
